# Sleep in the academic sphere: identifying sleep profiles and their influencing factors using latent profile analysis in German university students

**DOI:** 10.1186/s40359-025-03280-0

**Published:** 2025-08-12

**Authors:** Johanna M. Schmickler, Simon Blaschke, Filip Mess, Nils Olson, Barbara Reiner, Thorsten Schulz, Julian Friedrich

**Affiliations:** 1 Associate Professorship of Didactics in Sport and Health, Department Health and Sport Sciences, TUM School of Medicine and Health, Technical University of Munich, Am Olympiacampus 11, 80809 Munich, Germany; 2 Chair of Preventive Pediatrics, Department Health and Sport Sciences, TUM School of Medicine and Health, Technical University of Munich, Munich, Germany

**Keywords:** Person-centered approach, Health promotion, Health services, Higher education

## Abstract

**Background:**

Poor sleep quality is a prevalent issue among university students, raising concerns for both individual well-being and public health. While the heterogeneity of sleep quality has been recognized, research examining distinct sleep quality subtypes and their influencing factors is still developing. This study aimed to explore sleep quality profiles among German university students and identify socio-demographic, health-related, and academic factors associated with profile membership.

**Methods:**

A total of 1,526 university students from various academic disciplines participated in the study. Data were collected via an online questionnaire, including the Pittsburgh Sleep Quality Index, Perceived Stress Scale, Utrecht Work Engagement Scale for Students, and questions on socio-demographic, health-related, and academic factors. Latent profile analysis and multinomial logistic regression were used to analyze the data.

**Results:**

We identified four distinct sleep quality profiles among our participants: the *Average Sleep Profile* (78.5%), the *Insomnia Risk Profile* (8.2%), the *Above Average Profile* (7.2%), and the *Medicated Sleepiness Profile* (6.1%). Prolonged sleep onset latency and daytime sleepiness were common across most profiles, indicating that these are widespread sleep-related issues among students. Female students (*OR* = 2.75, *p* < 0.001) and those with higher stress levels (*OR* = 1.09, *p* < 0.001) were more likely to belong to the *Insomnia Risk Profile*. Older students (aged 23 years or above) (*OR* = 1.88, *p* = 0.030), those enrolled in State examination programs (*OR* = 5.73, *p* = 0.016) as well as students experiencing higher stress (*OR* = 1.08, *p* < 0.001) and academic workload (*OR* = 1.01, *p* = 0.022), had an increased likelihood of belonging to the *Medicated Sleepiness Profile*. Students reporting better subjective health status were less likely to be assigned to profiles characterized by maladaptive sleep patterns.

**Conclusion:**

These findings underscore the variability in sleep quality among university students, offering insights for the development of tailored preventive interventions. Future sleep promotion programs should consider individual differences when designing strategies to address the diverse sleep challenges faced by students.

**Supplementary Information:**

The online version contains supplementary material available at 10.1186/s40359-025-03280-0.

## Background

Sleep quality is a widely recognized concept in both sleep medicine and sleep research. Although it remains poorly defined [1], sleep quality can be understood as a multidimensional construct, composed of both subjective and objective attributes, that are used to distinguish between good and poor sleepers [2]. Key components of sleep quality include sleep duration, sleep latency, sleep efficiency, subjective sleep quality, sleep disturbances, daytime dysfunction, and the use of sleep medicine [3].

Unfortunately, poor sleep quality is commonly observed across the lifespan, with sleep problems emerging for some in the early twenties as individuals progress from adolescence to young adulthood [4]. Within the transition from high school to higher education, young adults typically experience significant changes at both the individual level (e.g., increased independence and biological processes) and contextual level (e.g., new living arrangements, changes in daily routines, and social dynamics) [5]. These changes require students to navigate the complex interplay of academic responsibilities, social commitments, and personal well-being, making them vulnerable to developing poor sleep quality. Numerous studies have raised alarm, revealing that up to one-third of university students obtain less than the recommended 7–9 h of sleep within a 24-hour period for young adults and adults [6–9]. In addition, nearly half are considered poor sleepers, experiencing symptoms such as difficulties falling asleep, frequent awakenings after sleep onset, and early morning awakenings [7, 10]. This is particularly concerning, as poor sleep quality can result in a wide range of immediate and long-term adverse health outcomes, such as cognitive performance deficits, increased cardiovascular risks, and psychological distress [11]. The directionality of the relationship between sleep and behavior and psychological functioning remains a topic of ongoing debate and emerging evidence suggests a bidirectional relationship, where poor sleep quality can contribute to the development of adverse health behaviors and mental health problems and can result from these challenges [12–15].

The factors influencing sleep quality in university students are complex. Researchers have focused on understanding the unique determinants of sleep quality within this population. For example, older age (aged 23 years or above), dormitory environment, low socio-economic status, high exhaustion levels, depression and anxiety symptoms, elevated stress, poor academic performance, and sedentary behavior have been associated with poor sleep quality [10, 16–18]. Moreover, differences in sleep quality among students from different academic disciplines may be influenced by variations in workload intensity, stress levels, attitudes toward sleep, and sleep hygiene practices [10, 19]. This variance may result in certain students being at greater risk of poor sleep quality than others.

Research investigating sleep quality among students has largely employed a variable-centered approach using methods such as correlation and regression analysis that treat students as a homogeneous group. However, building on prior studies [20–22] it is likely that students exhibit heterogeneity in sleep quality, making person-centered approaches essential to identify distinct sleep quality profiles and investigate key factors that are decisive for these variations. Moreover, current interventions [23] often adopt a generalized *one-size-fits-all* approach, overlooking the multidimensional nature of sleep quality and the interplay of individual determinants that influence sleep quality [24]. This approach not only limits their effectiveness but also reduces the potential for long-term success of interventions in this field. Although person-centered approaches have identified distinct student health profiles [25, 26] research investigating sleep profiles in students remains limited, with only a few studies addressing this gap [27–30].

Carpi et al. (2022) investigated insomnia profiles in university students with clinically relevant insomnia symptoms, identifying a five-profile model. They found that profiles with higher insomnia severity were associated with worse mental health and lower health-related quality of life. However, no significant differences in other health behaviors, such as physical exercise, tobacco use, and alcohol consumption were observed between the insomnia profiles. Chen et al. (2023) explored sleep disturbances in a large sample of Chinese college students. They identified three profiles and demonstrated that male students, sophomores, and those from families with poor parental marital status were more likely to experience sleep disturbances. In a study of medical students, Yu et al. (2023) examined latent profiles of health-related quality of life, sleep quality, chronotype, and internet addiction, finding that students with the best sleep quality also reported higher health-related quality of life and lower levels of internet addiction. Similarly, Zhang et al. (2024) investigated sleep quality profiles among medical students, finding that 74% were assigned to the *good sleep group*, while 24% were in the *daytime dysfunction group*, and 2% in the *sleep disorder group*. Membership in the latter two groups was linked to poorer interpersonal relationships, compromised health, evening chronotype, and fewer health-promoting lifestyle habits. Although promising, studies investigating students’ sleep quality profiles have largely followed a disease-oriented approach, focusing on insomnia profiles in relation to psychological distress, chronotype, or health-related quality of life. Moreover, existing research is mostly limited to specific socio-cultural regions and academic disciplines.

Consequently, a more comprehensive understanding of sleep quality, especially its heterogeneity among university students, is needed. Although previous studies have identified critical determinants of students’ sleep quality, the precise roles of these biopsychosocial factors in shaping distinct sleep quality profiles require further investigation. This knowledge is crucial for designing personalized interventions that adopt a more holistic health-oriented approach, focusing on promoting overall well-being rather than solely addressing sleep problems [31]. Based on these premises, the aim of the current study is (1) to identify distinct profiles of sleep quality among university students from different academic disciplines, and (2) to characterize these profiles based on socio-demographic, health-related, and academic factors.

## Methods

### Participants and procedure

Data for this cross-sectional study were collected between July and August 2021 via an online survey that was hosted on Enterprise Feedback Suite Survey Unipark (Tivian XI GmbH, Cologne, Germany). A total of 45,812 students were contacted via their university email addresses and invited to participate. Individuals aged 18 years or older were eligible to participate. Participation was voluntary, and written consent was required from all participants. The study received full approval from the local ethics committee’s Institutional Review Board and was conducted in accordance with the Declaration of Helsinki.

### Measures

#### Socio-demographics

Socio-demographic data were collected, including gender (female, male, non-binary) and age. Subjective social status was measured using the 10-point MacArthur Scale [32], with higher numbers indicating higher perceived subjective status regarding income, education, and employment.

#### Health-related factors

Self-rated health over the previous two months was assessed using a 10-point Likert scale, with higher scores indicating better perceived health. Perceived stress was measured using the Perceived Stress Scale (PSS-10) [33], a widely used self-report instrument consisting of 10 items rated on a 5-point frequency scale (0 = never; 4 = very often). The PSS-10 total score ranges from 0 to 40, with higher scores representing greater levels of perceived stress.

#### Academic factors

Academic background included study degree program and academic discipline and was assessed through dedicated questions. In this study, we distinguished between undergraduate and graduate students, as well as students in State examination programs, due to relevant differences in academic structure, institutional enrollment, and daily routines. At the university where this study was conducted, the majority of State examination students were enrolled in medicine and teacher training programs. In general, state examination candidates represent a distinct group commonly found in professional degree tracks such as law, medicine, or education, particularly within the German-speaking academic context. These programs often involve a higher proportion of practical training and differ structurally from other academic programs. While they include intermediate examinations, they result in a large, centralized regional State Examination at the end of the study period. Compared to other degree programs, these tracks require a higher degree of self-management, as the semester structure and study plans are often less standardized and more individually organized.

Program study satisfaction was evaluated with a single item, asking students to rate their satisfaction with their choice of the study program on a scale from 1 to 10, where higher scores indicate greater study satisfaction. Weekly study workload was assessed by asking participants how many hours per week they typically spend in lectures and seminars, and for preparation and review activities related to their studies. Student engagement was measured using the German short version of the Utrecht Work Engagement Scale, Student Version (UWES-9 S) [34]. The scale consists of three subscales (vigor, dedication, absorption) each containing three items rated on a 7-point frequency scale (0 = never; 6 = always). An overall mean score of the UWES-9 S was computed, with higher scores reflecting greater student engagement.

#### Sleep quality

Sleep quality was assessed using the German version of the Pittsburgh Sleep Quality Index (PSQI) [3]. The PSQI is a self-report questionnaire designed to evaluate sleep quality and disturbances over the past month, consisting of 19 items divided into seven components: subjective sleep quality, sleep latency, sleep duration, habitual sleep efficiency, sleep disturbances, use of sleep medication, and daytime dysfunction. Each component is rated on a scale from 0 to 3, and the scores are summed to produce a global score ranging from 0 to 21. A global PSQI score of 5 or higher indicates poor sleep quality, with high sensitivity (90%) and specificity (87%) for identifying sleep disorders across all age groups [3].

### Statistical analysis

Data analysis was conducted using R and the RStudio interface (Version 4.3.3, RStudio Inc., Boston, MA, USA). Ineligible cases and invalid questionnaire responses were removed from the data set [35]. Due to a small sample size students who indicated non-binary gender (*n* = 13) were excluded from statistical data analysis. However, their descriptive characteristics with regard to the data collected for this study are presented in the Supplementary, Table 5. In our study, 17% of the remaining participants presented over 30% missing values and were excluded (listwise deletion) based on descriptive analyses under the assumption of non-random missingness due to item nonresponse [36]. For valid cases with missing data (1.2% of the sample), values were imputed using a weighted average based on the *k*-nearest neighbor (*k-*NN) algorithm (*k* = 10). Multivariate outliers were detected using Mahalanobis distance with a *p*-value of 0.001 used as a cutoff resulting in the removal of 45 cases and a total inclusion of *n* = 1,526 participants.

Latent profile analysis (LPA) was conducted to understand patterns of sleep quality among students using the *mclust* package (version 6.1; [37]). The aim was to identify homogeneous subgroups (profiles) concerning the seven components of sleep quality within the PSQI. Prior to performing LPA, we calculated the Variance Inflation Factor (VIF) for each sleep component to assess the degree of multicollinearity. A VIF value greater than 5 was used as a threshold to indicate problematic multicollinearity [38].

Several fit indices were used to determine the optimal number of profiles, including the Akaike Information Criterion (AIC), Bayesian Information Criterion (BIC), sample size-adjusted Bayesian Information Criterion (aBIC), Bootstrapped Likelihood Ratio Test (BLRT), and entropy, which reflects the model’s ability to distinguish well-separated profiles. Lower AIC, BIC, and aBIC values, significant BLRT *p*-values, and higher entropy scores were considered indicators of a better model fit [39]. We also prioritized profile interpretability, ensuring that each profile contained at least 5% of the sample [40].

After identifying and describing the profiles, we conducted a multinomial logistic regression analysis to determine whether socio-demographic, health-related, or academic factors predicted profile membership. Odds ratios with 95% confidence intervals and *p*-values were used to assess factor influences on profile membership [41]. The overall model accuracy was calculated as the proportion of correct predictions by comparing generated classifications to actual classifications. A one-way analysis of variance (ANOVA) was conducted to assess statistical differences in the means of categorical variables across the identified sleep quality profiles. A two-sided *p*-value of *p* < 0.05 was considered statistically significant, with a 95% confidence interval.

## Results

### Sample characteristics

Of the 2,284 individuals who initially started the survey (response rate: 5%), a final sample of 1,526 university students remained for this study after data preprocessing. Mean age of the total sample was *M* = 22.85 (*SD* = 3.14) years. Among the participants, 751 (49%) were men and 775 (51%) were women. The majority were undergraduate students (51%), with notable representation from students in Informatics and Technology (24%) and Engineering and Design (22%).

### Identification of sleep quality subtypes

The seven components of the PSQI were used as indicators to analyze student sleep quality profiles through LPA. VIF analysis revealed values within acceptable thresholds. The fit indices for the evaluated LPA models are shown in Table 1. The analysis using an ellipsoidal, equal volume, and equal shape (EEV) model indicated that the nine-profile solution had the lowest BIC (BIC = − 7855). However, compared to the four-profile solution the nine-profile solution showed a higher AIC, lower BLRT, lower entropy, and several profiles smaller than 5% of the sample. Considering the above, including the relative sizes of the four profiles and their conceptual relevance, the four-profile solution was selected as the optimal model. The predicted allocation probability was higher than 0.8 in 92% of the sample.

**Table 1:** Fit indices for the models examined with latent profile analysis

Profile	AIC	BIC	aBIC	Entropy	BLRT	BLRT *p*-value
**1**	16,890	−17,077	−17,084	0.946		
**2**	13,390	−13,731	−13,746	0.999	3,559	< 0.001
**3**	8,882	−9,378	−9,400	0.936	4,565	< 0.001
**4**	8,827	−9,477	−9,507	0.995	113	< 0.001

Note. AIC, Akaike Information Criterion; BIC, Bayesian Information Criterion; aBIC, sample size-adjusted Bayesian Information Criterion; BLRT, Bootstrap Likelihood Ratio Test

### Characterization of sleep quality profiles

Tables 2 and 3 present the overall sample characteristics across the four sleep quality profiles, including socio-demographic, health-related, and academic factors, as well as sleep characteristics and PSQI components. Results from the ANOVA examining statistical differences between the sleep quality profiles are provided in the supplementary material (Table 6).

Based on the observed sleep quality attributes and most remarkable characteristics, descriptive labels were added: (1) The *Average Sleep Profile* (78.5% of the sample) was the largest profile, with sleep quality component values closely aligning with the respective overall means. Moreover, students in this profile reported fairly good subjective sleep quality, satisfactory sleep efficiency, and below-average sleep duration, sleep disturbances and daytime functionality. However, they experienced challenges with sleep latency (*M* = 1.32, *SD* = 0.09) compared to the *Above Average Sleep Profile* (*M* = 0.00, *SD* = 0.00). Additionally, students indicated no use of sleep medication. (2) The *Insomnia Risk Profile* (8.2% of the sample) included students who reported higher values across several sleep quality components, reflecting overall poorer sleep quality characteristics. This profile exhibited the highest values among all profiles for sleep latency (*M* = 2.08, *SD* = 0.80), indicating it usually took students between 31 and 60 min to fall asleep at night. Moreover, students in this profile showed the highest levels of sleep disturbances (*M* = 2.00, *SD* = 0.00) and daytime dysfunction (*M* = 1.98, *SD* = 0.62), both occurring once or twice a week. (3) The *Above Average Sleep Profile* (7.2% of the sample) included participants who reported considerably low sleep problems across all sleep quality components, except for sleep duration (*M* = 0.40, *SD* = 0.56) and daytime dysfunction (*M* = 1.55, *SD* = 0.81). Both of these components were higher than those reported by students in the *Average Sleep Profile*. In line with the *Average Sleep Profile*, students in the *Above Average Sleep Profile* did not report using sleep medication (*M* = 0.00, *SD* = 0.00). (4) The *Medicated Sleepiness Profile* (6.1% of the sample) made up for those students who reported considerable sleep quality complaints and indicated the use of sleep medication (*M* = 0.90, *SD* = 0.77). In addition, this profile demonstrated the highest values for sleep duration (*M* = 0.98, *SD* = 0.90), indicating that students usually got between 6 and 7 h of sleep.

In addition to the PSQI-based sleep quality components used to construct the latent profiles, we separately assessed specific sleep timing variables, including bedtime, wake time, total sleep duration, and sleep latency to provide complementary insights into the sleep behavior associated with each sleep quality profile. Overall, these findings reflect existing research indicating that an increased proportion of students demonstrate a preference for evening chronotype [42]. The *Average Sleep Profile* showed the longest total sleep duration. In contrast, the *Insomnia Risk Profile* reported the shortest sleep duration despite having a typical bedtime close to the overall sample average. This profile was characterized by notably high sleep latency, averaging 47 min per night. The *Above Average Sleep* and *Medicated Sleepiness Profiles* exhibited differing sleep characteristics: while students in the *Above Average Profile* reported a very low sleep latency and average sleep duration, students in the *Medicated Sleepiness Profile* slept for a shorter duration. However, both profiles showed a tendency toward a later circadian preference, with later bedtimes and earlier wake times compared to the other two profiles.

**Table 2:** Socio-demographic characteristics and academic background

Variables	Total Sample (*N* = 1,526)	Average Sleep Profile (*n* = 1,198)	Insomnia Risk Profile (*n* = 125)	Above Average Sleep Profile (*n* = 110)	Medicated Sleepiness Profile (*n* = 93)
**Gender** Male Female					
	751 (49%)	619 (52%)	29 (23%)	60 (55%)	43 (46%)
	775 (51%)	579 (48%)	96 (77%)	50 (45%)	50 (54%)
**Age group**					
18–22 years	750 (49%)	607 (51%)	56 (45%)	50 (45%)	37 (40%)
≥ 23 years	776 (51%)	591 (49%)	69 (55%)	60 (55%)	56 (60%)
**Degree**					
Undergraduate	775 (51%)	616 (51%)	63 (50%)	51 (46%)	45 (48%)
Graduate	601 (39%)	473 (40%)	48 (38%)	45 (41%)	35 (38%)
State Examination	150 (10%)	109 (9%)	14 (11%)	14 (13%)	13 (14%)
**Academic Discipline**					
Business Administration	109 (7%)	83 (7%)	9 (7%)	9 (8%)	8 (9%)
Medicine	129 (8%)	97 (8%)	13 (10%)	10 (9%)	9 (10%)
Sport and Health Science	125 (8%)	100 (8%)	11 (9%)	9 (8%)	5 (5%)
Informatics and Technology	369 (24%)	312 (26%)	17 (14%)	21 (19%)	19 (20%)
Natural Sciences	194 (13%)	149 (12%)	19 (15%)	10 (9%)	16 (17%)
Life Sciences	130 (9%)	94 (8%)	14 (11%)	15 (12%)	9 (10%)
Social Sciences and Technology	55 (4%)	42 (4%)	6 (5%)	4 (4%)	3 (3%)
Engineering and Design	341 (22%)	270 (23%)	29 (23%)	28 (25%)	14 (15%)
Other	74 (5%)	51 (4%)	7 (6%)	6 (5%)	10 (11%)

*Notes.N* = 1,526; values are expressed as frequencies and percentages

**Table 3:** Socio-demographic, health-related, academic factors, sleep characteristics and PSQI component characteristics

Variables	Total Sample (*N* = 1,526)	Average Sleep Profile (*n* = 1,198)	Insomnia Risk Profile (*n* = 125)	Above Average Sleep Profile (*n* = 110)	Medicated Sleepiness Profile (*n* = 93)
**Subjective Social Status**	6.31 ± 1.78	6.36 ± 1.77	5.96 ± 1.86	6.61 ± 1.74	5.83 ± 1.79
**Subjective Health**	5.98 ± 2.37	6.22 ± 2.29	4.35 ± 2.14	6.37 ± 2.54	4.63 ± 2.24
**Perceived Stress**	20.73 ± 7.17	20.00 ± 6.97	26.30 ± 5.63	18.80 ± 7.35	25.40 ± 6.12
**Study Satisfaction**	7.72 ± 1.99	7.80 ± 1.94	7.08 ± 2.25	8.19 ± 1.83	7.06 ± 2.15
**Weekly Study Workload**, *hours*	35.77 ± 17.93	35.20 ± 17.20	37.90 ± 20.00	35.60 ± 18.40	40.60 ± 22.00
**Study Engagement**	3.02 ± 1.09	3.06 ± 1.07	2.64 ± 1.06	3.30 ± 1.13	2.59 ± 1.07
**Bedtime**, *HH: MM ± minutes*	11:44 ± 75.0	11:42 ± 74.5	11:37 ± 64.5	00:04 ± 75.4	00:03 ± 87.8
**Wake Time**, *HH: MM ± minutes*	08:04 ± 81.7	08:06 ± 80.8	08:10 ± 88.2	07:45 ± 75.0	07:45 ± 88.4
**Sleep Duration**, *hours*	7.26 ± 0.98	7.38 ± 0.88	6.86 ± 1.08	7.15 ± 1.11	6.33 ± 1.17
**Sleep Latency**, *minutes*	29.87 ± 28.53	29.20 ± 27.70	47.00 ± 32.30	7.79 ± 4.44	41.40 ± 30.90
**PSQI Global Score**	5.90 ± 2.66	5.49 ± 2.15	9.43 ± 2.42	3.46 ± 1.81	9.34 ± 2.63
**PSQI Component Scores**					
Subjective Sleep Quality	1.21 ± 0.65	1.15 ± 0.60	1.70 ± 0.60	0.83 ± 0.76	1.71 ± 0.64
Sleep Latency	1.31 ± 0.96	1.32 ± 0.90	2.08 ± 0.80	0.00 ± 0.00	1.80 ± 0.88
Sleep Duration	0.27 ± 0.52	0.17 ± 0.40	0.55 ± 0.64	0.40 ± 0.56	0.98 ± 0.90
Habitual Sleep Efficiency	0.48 ± 0.74	0.42 ± 0.67	0.98 ± 1.02	0.09 ± 0.32	0.99 ± 0.95
Sleep Disturbances	1.03 ± 0.37	0.97 ± 0.17	2.00 ± 0.00	0.59 ± 0.56	0.99 ± 0.10
Use of Sleep Medications	0.07 ± 0.31	0.00 ± 0.00	0.14 ± 0.42	0.00 ± 0.00	0.90 ± 0.77
Daytime Functionality	1.53 ± 0.73	1.45 ± 0.70	1.98 ± 0.62	1.55 ± 0.81	1.98 ± 0.74

*Notes. N* = 1,526; values are expressed as mean ± standard deviation. PSQI components range from 0 to 3, with higher values indicating more maladaptive sleep quality outcomes

### Multinomial logistic regression analysis

Table 4 presents the multinomial logistic regression analysis results. The *Average Sleep Profile* served as a reference profile. By accounting for confounding factors, regression analysis allowed for the evaluation of the relative effect of socio-demographic, health-related, and academic factors on the likelihood of belonging to each sleep quality profile. In our study, the overall predicted model accuracy was 78.7%.

Compared to the *Average Sleep Profile*, female students (*OR* = 2.75, *p* < 0.001) and students with higher perceived stress levels (*OR* = 1.09, *p* < 0.001) had significantly higher odds of being assigned to the *Insomnia Risk Profile*. Conversely, students reporting better subjective health (*OR* = 0.80, *p* < 0.001) were less likely to be assigned to this profile.

In comparison to the *Average Sleep Profile*, students enrolled in a State examination program (e.g., medical students and students in a teacher training program) demonstrated significantly increased odds of being classified within the *Above Average Sleep Profile* (*OR* = 3.82, *p* = 0.042).

Relative to the *Average Sleep Profile*, older students (aged 23 years or older; *OR* = 1.88, *p* = 0.030) along with students enrolled in a State examination program (*OR* = 5.73, *p* = 0.016) were significantly more likely to be assigned to the *Medicated Sleepiness Profile*. Additionally, higher weekly workloads (*OR* = 1.01, *p* = 0.022) and elevated perceived stress levels (*OR* = 1.08, *p* < 0.001) were associated with an increased likelihood of being categorized within the *Medicated Sleepiness Profile*. However those reporting better subjective health (*OR* = 0.87, *p* = 0.024) were significantly less likely to be assigned to this profile.

**Table 4:** Results of the multinomial logistic regression analysis

Variables	Insomnia Risk Profile (*n* = 125)		Above Average Sleep Profile (*n* = 110)		Medicated Sleepiness Profile (*n* = 93)	
	OR [95% CI]	*p*	OR [95% CI]	*p*	OR [95% CI]	*p*
*Intercepts*	**0.02 [0.00**,** 0.14]**	**< 0.001**	**0.05 [0.01**,**0.34]**	**0.002**	**0.03 [0.00**,** 0.23]**	**0.001**
**Gender**						
Male	1.00		1.00		1.00	
Female	**2.75 [1.72**,** 4.41]**	**< 0.001**	0.82 [0.53, 1.26]	0.365	0.98 [0.61, 1.57]	0.920
**Age group**						
18–22 years	1.00		1.00		1.00	
≥ 23 years	1.36 [0.81, 2.26]	0.242	1.18 [0.69, 2.00]	0.554	**1.88 [1.06**,** 3.33]**	**0.030**
**Subjective Social Status**	0.96 [0.86, 1.07]	0.432	1.05 [0.94, 1.17]	0.382	0.93 [0.83, 1.05]	0.264
**Subjective Health**	**0.80 [0.72**,** 0.90]**	**< 0.001**	0.95 [0.85, 1.07]	0.407	**0.87 [0.78**,** 0.98]**	**0.024**
**Perceived Stress**	**1.09 [1.05**,** 1.14]**	**< 0.001**	0.98 [0.95, 1.02]	0.431	**1.08 [1.04**,** 1.13]**	**< 0.001**
**Degree**						
Undergraduate	1.00		1.00		1.00	
Graduate	0.87 [0.50, 1.50]	0.607	0.95 [0.53, 1.68]	0.851	0.76 [0.41, 1.39]	0.367
State Examination	0.79 [0.14, 4.41]	0.784	**3.82 [1.05**,** 13.86]**	**0.042**	**5.73 [1.39**,** 23.67]**	**0.016**
**Academic Discipline**						
Business Administration	1.00		1.00		1.00	
Medicine	1.45 [0.21, 9.95]	0.707	0.24 [0.05, 1.17]	0.077	**0.16 [0.03**,** 0.93]**	**0.041**
Sport and Health Science	1.02 [0.37, 2.81]	0.973	0.71 [0.24, 2.07]	0.531	0.39 [0.10, 1.49]	0.169
Informatics and Technology	0.63 [0.26, 1.56]	0.320	0.59 [0.26, 1.37]	0.222	0.62 [0.25, 1.52]	0.296
Natural Sciences	1.26 [0.51, 3.10]	0.621	0.62 [0.24, 1.61]	0.323	1.08 [0.42, 2.76]	0.874
Life Sciences	0.97 [0.37, 2.51]	0.944	1.45 [0.58, 3.62]	0.430	0.80 [0.28, 2.27]	0.673
Social Sciences and Technology	1.43 [0.44, 4.41]	0.553	0.74 [0.20, 2.71]	0.653	0.65 [0.15, 2.78]	0.557
Engineering and Design	1.04 [0.45, 2.41]	0.929	0.92 [0.41, 2.04]	0.833	0.49 [0.19, 1.26]	0.140
Other	1.77 [0.58, 5.42]	0.318	1.16 [0.38, 3.49]	0.798	2.35 [0.81, 6.77]	0.114
**Study Satisfaction**	0.99 [0.88, 1.12]	0.900	1.08 [0.93, 1.25]	0.303	1.03 [0.90, 1.18]	0.651
**Weekly Study Workload**	1.00 [0.99, 1.01]	0.677	1.00 [0.99, 1.01]	0.726	**1.01 [1.00**,** 1.03]**	**0.022**
**Study Engagement**	0.98 [0.76, 1.27]	0.878	1.11 [0.86, 1.43]	0.405	0.83 [0.62, 1.11]	0.214

Note. Bold numbers indicate significant findings with a *p*-value < 0.05

## Discussion

This study explored sleep quality profiles in university students across various academic disciplines and examined the influence of socio-demographic, health-related, and academic factors on profile membership.

### Characterization of sleep quality profiles

Based on the multidimensional construct of sleep quality [3], this study identified four distinct profiles: the *Average Sleep Profile*, the *Insomnia Risk Profile*, the *Above Average Sleep Profile*, and the *Medicated Sleepiness Profile*. The number of profile solutions is in between previous findings of three [27, 28, 30] and five sleep profiles [29]. The *Average Sleep Profile* (78.5%) included students with generally typical sleep quality, moderate sleep latency, and no use of sleep medication. The *Insomnia Risk Profile* (8.2%) captured students, more often female and highly stressed, who reported poor sleep across all components, especially difficulties falling asleep and high daytime dysfunction. The *Above Average Sleep Profile* (7.2%) reflected students, often in State examination programs, with few sleep complaints and overall good sleep quality. Lastly, the *Medicated Sleepiness Profile* (6.1%) consisted primarily of students (aged 23 years or above) or those facing high stress and academic demands, characterized by reported sleep medication use, shorter sleep duration, and increased daytime dysfunction.

Compared to prior studies [7, 29], students in our sample showed better sleep patterns on several PSQI components but reported poorer subjective sleep quality and increased daytime sleepiness. However, in contrast to findings from a study conducted among Chinese college students [43], our participants exhibited generally worse sleep characteristics, with the exception of longer sleep duration and less daytime sleepiness. Except for the *Above Average Sleep Profile*, all sleep quality profiles identified in the current study reported delayed sleep onset latency, with most students taking around 30 or more minutes to fall asleep – exceeding the average sleep latency of 12 min reported in a review of healthy adult cohorts [44]. Prolonged sleep onset latencies in student cohorts have also been frequently reported in earlier studies [7, 45] and were linked to poorer overall sleep quality and mental health outcomes, including symptoms of anxiety and depression. Additionally, longer sleep latency was found to be significantly more common among female students [7, 45]. While differences in sleep quality components between this and previous studies may be partly explained by demographic factors, such as a higher proportion of female students [7, 29], a younger average age [43], or the focus on specific academic disciplines [29], the present study offers important insights into student’s sleep quality. In particular, the findings underscore that prolonged sleep latency and excessive daytime sleepiness are common and significant challenges affecting overall sleep quality among university students.

Compared to other studies [27–29] that incorporated additional variables, such as health-related quality of life or insomnia symptoms, to form sleep profiles, our findings are most directly comparable to those of Zhang et al. (2024), who similarly used only PSQI components as indicator variables in their profile analysis. However, notable differences in sleep characteristics emerge when analyzing the largest profiles from both studies. In Zhang et al. (2024), who focused exclusively on medical students, the largest profile, the *good sleep group*, showed the lowest scores across all PSQI components, indicating overall good sleep quality. In contrast, the largest profile in the present study, the *Average Sleep Profile*, revealed common challenges with prolonged sleep latency, frequent sleep disturbances, and increased daytime sleepiness, pointing to more heterogeneous and nuanced sleep difficulties. Despite these differences, both studies identified problematic daytime dysfunction across all sleep quality profiles. In the PSQI daytime dysfunction includes difficulties staying awake while driving, eating, or engaging in social activities, as well as reduced enthusiasm for completing tasks. These issues are likely linked to high academic demands and elevated stress levels. By including both undergraduate and graduate students from a variety of academic disciplines, our study contributes to the existing research and demonstrates that problematic daytime dysfunction is not limited to medical students but widespread across the broader student population.

### Characterization of sleep quality profile membership

This study aimed to characterize sleep quality profile membership based on socio-demographic (e.g., gender, age group, and subjective social status), health-related (e.g., subjective health and perceived stress), and academic factors (e.g., degree, academic discipline, study satisfaction, weekly study workload, and study engagement) through multinomial logistic regression analysis. The *Average Sleep Profile* was the largest profile and served as the reference category in our regression analysis, which identified several significant risk factors influencing profile membership.

*Insomnia Risk Profile* – Female students were significantly more likely to be assigned to the *Insomnia Risk Profile*. Insomnia is typically characterized by difficulties initiating or maintaining sleep accompanied by daytime impairments, and is known to show a higher prevalence among older adults and consistent female predominance across the lifespan [46]. This is supported by our study findings and previous research [7, 44, 45], which indicate that female students are more likely to report poor sleep quality, characterized by symptoms such as daytime dysfunction, prolonged sleep onset, lower sleep efficiency, increased sleep disturbances, and greater use of sleep aids. While the exact reasons for sex differences in sleep remain under discussion, hormonal fluctuations are considered a contributing factor. For instance, toward the end of the menstrual cycle, women experience decreased sleep efficiency, shorter total sleep time, and more frequent awakenings [47]. Moreover, studies suggest that women report a greater physiological need for sleep compared to men, yet this need may be unmet due to high academic demands and extracurricular obligations [48]. As a result, women are more prone to excessive daytime sleepiness and more likely to rate their overall sleep quality as poor. Additionally, the mismatch between biological sleep needs and the timing of social and academic activities, often referred to as social jetlag, may disproportionately affect young women. This misalignment is associated with sleep deprivation and has been linked to increased levels of depressive symptoms [49].

In the present study, students with higher self-reported health were significantly less likely to fall into the *Insomnia Risk Profile*. This aligns with previous research [30] showing that students who rated their health as poor were more often grouped into the *sleep disorder* and *daytime dysfunction* groups rather than the *good sleep* group. Better self-rated health may reflect a generally healthier lifestyle, including more balanced routines, regular physical activity, and effective stress management, which in turn can positively influence sleep quality. Students with higher health consciousness and awareness may be more likely to engage in behaviors that promote good sleep hygiene and overall well-being. This pattern was also observed in a study investigating behavioral health risk profiles in a student cohorts in Great Britain, where healthier lifestyle patterns and better sleep were associated with assignment to the *high physically active and health-conscious* cluster [50].

Additionally, the findings demonstrated that the perceived level of stress significantly predicted profile membership indicating that students reporting higher stress levels were more likely to be belong to the *Insomnia Risk Profile.* This is consistent with research, which highlights the strong interconnection between stress and sleep quality in student populations [51]. Although the causal relationship remains to be fully understood, these sleep problems may be driven by sleep-interfering factors such as heightened pre-sleep arousal, slow recovery from academic stress, heavy workloads, and emotional conflicts [52].

*Medicated Sleepiness Profile* – In our study, older students (aged 23 years or older) were significantly more likely to be assigned to the *Medicated Sleepiness Profile*. This aligns with prior research indicating higher prevalence of sleep aid use among older university students [53]. Compared to younger students or first-year undergraduates, more mature students may face increased academic demands and pressure related to career planning and post-graduation employment. These stressors could contribute to difficulties in initiating and maintaining sleep. Notably, students in the *Medicated Sleepiness Profile* also reported the highest weekly workload, high levels of perceived stress, and the lowest levels of study satisfaction and academic engagement. These patterns suggest that the use of sleep medication may be used as a coping strategy to manage overwhelming academic pressures and personal demands.

Although students enrolled in State examination programs were significantly more likely to be categorized within the *Above Average Sleep Profile* and the *Medicated Sleepiness Profile*, regression analysis revealed that medical students, who also fall under State examination programs, were less likely to belong to the *Medicated Sleepiness Profile*. This discrepancy may be attributed to medical students’ greater awareness of the potential risks and side effects associated with sleep medications, which could lead to more cautious or limited use compared to students in other academic disciplines. Therefore, it is plausible that students assigned to the *Medicated Sleepiness Profile* within State examination programs were predominantly teacher training students, rather than medical students. Given the relatively small sample size of students in State examination programs and the scarcity of existing research focused on this cohort, further investigations is needed to better understand these patterns and their implications.

Students who reported higher academic workloads were significantly more likely to be assigned to the *Medicated Sleepiness Profile.* This findings aligns with research conducted in the general population demonstrating that high work demands and increased mental work hours are strong risk factors for sleep disturbances, increased daytime sleepiness, difficulties awakening, and overall reduced sleep quality [54, 55]. These parallels suggest that academic demands may mirror occupational stressors in their impact on sleep quality, underscoring the importance of addressing workload-related pressures in student populations.

### Implications for research and practice

The findings have important implications for developing targeted interventions to improve student sleep quality. For the *Average Sleep Profile*, which comprised the majority of the sample, prolonged sleep onset and daytime sleepiness emerged as prevalent issues. This highlights the potential benefit of educational initiatives that promote behavioral strategies to support healthy sleep initiation and enhance daytime alertness. Such interventions might include text message campaigns providing practical tips [56], such as limiting caffeine intake in the evening, avoiding binge drinking [57], and engaging in relaxing pre-sleep activities like reading or meditation outside of bed to reinforce a positive association between the bed and falling asleep quickly [58]. In addition, incorporating outdoor teaching methods could help increase daytime exposure to natural light, enhancing student alertness, mood, and overall well-being [59] and has been shown to reduce sleep onset latency and improve sleep quality [58, 60]. Based on our findings, two profiles appear to be particularly in need of intervention due to their poor sleep quality outcomes. The first profile, *Insomnia Risk Profile*, primarily included female students and those with elevated stress levels. This profile was marked by significant daytime sleepiness, prolonged sleep onset, and difficulty maintaining sleep. Individualized interventions tailored to these students’ needs could focus on the interplay between sleep and mental health, addressing topics like stress management, mindfulness, and resilience. Additionally, considering the more than two thirds female membership, a three-fold approach improving sleep education, sleep duration and sleep quality with content tailored to the specific needs of female students may be especially beneficial. This includes addressing the influence of hormonal fluctuations on sleep. While research suggest that women may have a greater need for sleep than men [61], enhancing sleep-related health literacy can empower students to better understand and manage their sleep patterns. The second profile, *Medicated Sleepiness Profile*, was more likely to include older students and those with high workloads and stress levels. This group reported poor subjective sleep quality, prolonged sleep latency, shorter sleep duration, and the use of sleep medication. To address their needs, interventions focusing on stress management and strategies to reduce workload, such as effective time management, could improve sleep quality and overall well-being, especially as they prepare to enter the workforce. In addition, behavior change strategies are needed to reduce reliance on sleep aids, which should be considered a temporary measure for sleep problems. While the PSQI does not differentiate between types of sleep medication, it remains unclear whether students use prescribed or over-the-counter products. Future research could incorporate specific questions regarding both stimulant and sleep medication use to better assess the types and patterns of substances students take. Moreover, educational campaigns that raise awareness about the appropriate use and potential risks of both types of sleep medication, alongside non-pharmacological alternatives, are warranted, especially given students’ limited knowledge in this area [62]. Moreover, universities could enhance support services for students nearing graduation by helping them find jobs and offering resources for older students, such as affordable childcare, to reduce their stress and improve their overall academic experience.

Addressing the unique needs of distinct sleep quality profiles necessitates moving beyond traditional variable-oriented approaches, which have primarily focused on population-wide interventions. Although these approaches have contributed to the development of various intervention strategies to improve sleep among students [63], there is an increasing need for research that offers guidance for individualized and adaptive interventions. Person-centered approaches, such as LPA, have the potential to address the multifaceted and dynamic factors that affect sleep quality in young adults within higher education. This approach represents an essential foundation for developing group-based, needs-oriented interventions, rather than relying solely on individualized strategies, aligning with the integrative precision prevention framework [64]. Such interventions could be integrated into university health promotion initiatives and made accessible to students.

### Limitations

Some limitations of this study should be considered. First, data were collected from a single academic institution in Germany, which limits the generalizability of our findings. Additionally, a response bias might exist as only 5% of all students participated in the study. Although our sample included an equal representation of male and female students, this does not align with the university’s actual gender distribution (64% male students). Possibly, our sample may not fully represent the broader student population, and the findings may not generalize to students from other socio-demographic backgrounds and regions. Additionally, ethnicity data were not collected in the questionnaire, preventing a more detailed analysis of its potential impact on the results. This is an important limitation, as prior research suggests that ethnicity can be a relevant factor influencing sleep outcomes [65]. Data collection took place between July and August 2021, during a period that was primarily conducted as an online semester due to the impact of the COVID-19 pandemic. This unique context may have influenced study-related outcomes. Compared to studies conducted among German university students prior to the global pandemic [66, 67], our sample reported lower levels of study satisfaction, higher stress, and reduced study engagement. Accordingly, future research using post-pandemic data is needed to examine whether the identified sleep profiles and their associated factors remain stable or have shifted following the return to in-person lectures and campus life. Regarding the methodology, profile membership was determined through a retrospectively self-reported questionnaire based on a narrow set of sleep quality components. This reliance on self-reported data, without the inclusion of objective measures limits the precision of sleep assessment and may introduce bias. Additional information, such as sleep hygiene practices, sleep knowledge, and a more detailed assessment of students’ chronotype, could enhance the identification of sleep profiles, offering a more comprehensive understanding of students’ sleep patterns and improving the development of tailored interventions in this field. Beyond perceived stress levels, the current study did not examine additional psychological factors (e.g., personality types), behavioral factors (e.g., screen time) or student-specific variables (e.g., being a working student, class rank, or perceived teacher support), that may be helpful for interpreting sleep quality profiles.

## Conclusion

This was one of the first studies to investigate how academic factors (e.g., degree, academic discipline, study satisfaction, study workload, and study engagement) impact sleep quality profile membership. Four distinct sleep quality profiles were identified, varying by socio-demographic, health-related, and academic factors. These findings highlight the inter-individual variations in sleep characteristics within the student population, providing valuable insights for developing tailored preventive measures. Future interventions should consider individual needs, guiding stakeholders and university health promotion services to design strategies that effectively address the unique sleep challenges faced by students.

## Supplementary Information

Below is the link to the electronic supplementary material.

Supplementary Material 1

## Data Availability

The data supporting the conclusions of the current study are available from the corresponding author on reasonable request.

## Abbreviations

aBIC: Sample size-adjusted Bayesian Information Criterion
AIC: Akaike Information Criterion
BIC: Bayesian Information Criterion
BLRT: Bootstrapped Likelihood Ratio Test
LPA: Latent Profile Analysis
PSQI: Pittsburgh Sleep Quality Index
VIF: Variance Inflation Factor
