# Supplementary material for: Sleep in the academic sphere: identifying sleep profiles and their influencing factors using latent profile analysis in German university students
Source: BMC Psychol. 2025 Aug 12;13:907. doi: 10.1186/s40359-025-03280-0 (PMC12345099; doi:10.1186/s40359-025-03280-0)
Supplement: Supplementary file 1 — Supplementary Material 1 [file 40359_2025_3280_MOESM1_ESM.docx]

**Table 5** Descriptive characteristics of total sample and non-binary subsample reported as means ± standard deviation or frequencies and percentages.

| **Variables** | **Total Sample**  (*N* = 1,526) | **Non-Binary Sample**  (*N* = 13) |
| --- | --- | --- |
| **Age** | 22.85 ± 3.14 | 22.08 ± 2.47 |
| **Degree** |  |  |
| Undergraduate | 775 (51 %) | 7 (54 %) |
| Graduate | 601 (39 %) | 6 (46 %) |
| State Examination | 150 (10 %) | - |
| **Academic Discipline** |  |  |
| Business Administration | 109 (7 %) | - |
| Medicine | 129 (8 %) | - |
| Sport and Health Science | 125 (8 %) | - |
| Informatics and Technology | 369 (24 %) | 5 (38 %) |
| Natural Sciences | 194 (13 %) | 4 (31 %) |
| Life Sciences | 130 (9 %) | 1 (8 %) |
| Social Sciences and Technology | 55 (4 %) | 1 (8 %) |
| Engineering and Design | 341 (22 %) | 2 (15 %) |
| Other | 74 (5 %) | - |
| **Subjective Social Status** | 6.31 ± 1.78 | 6.31 ± 2.02 |
| **Subjective Health** | 5.98 ± 2.37 | 6.31 ± 2.56 |
| **Perceived Stress** | 20.73 ± 7.17 | 21.62 ± 5.03 |
| **Study Satisfaction** | 7.72 ± 1.99 | 7.38 ± 2.36 |
| **Weekly Study Workload**, *hours* | 35.77 ± 17.93 | 30.90 ± 15.36 |
| **Study Engagement** | 3.02 ± 1.09 | 2.55 ± 1.01 |
| **Sleep Duration***, hours* | 7.26 ± 0.98 | 7.31 ± 1.11 |
| **Sleep Latency***, minutes* | 29.87 ± 28.53 | 25.77 ± 15.39 |
| **PSQI Sum Score** | 5.90 ± 2.66 | 5.54 ± 2.96 |
| **PSQI Component Scores** |  |  |
| Subjective Sleep Quality | 1.21 ± 0.65 | 1.15 ± 0.38 |
| Sleep Latency | 1.31 ± 0.96 | 1.08 ± 0.76 |
| Sleep Duration | 0.27 ± 0.52 | 0.31 ± 0.63 |
| Habitual Sleep Efficiency | 0.48 ± 0.74 | 0.31 ± 0.85 |
| Sleep Disturbances | 1.03 ± 0.37 | 0.92 ± 0.28 |
| Use of Sleep Medications | 0.07 ± 0.31 | 0.23 ± 0.83 |
| Daytime Functionality | 1.53 ± 0.73 | 1.54 ± 0.78 |

**Table 6** Results of the Welch’s ANOVA and Games-Howell post-hoc test for differences between sleep quality profiles.

| **Variables** | **Average Sleep Profile**  **(AS)**  (*n* = 1,198) | **Insomnia Risk Profile**  **(IR)**  (*n* = 125) | **Above Average Sleep Profile (AA)**  (*n* = 110) | **Medicated Sleepiness Profile**  **(MS)**  (*n* = 93) | **F-Report** | **Games-Howell**  **Post-Hoc Test** |
| --- | --- | --- | --- | --- | --- | --- |
| **Age** | 22.80 ± 3.05 | 23.10 ± 3.50 | 23.10 ± 3.69 | 23.20 ± 3.01 | F(3, 201.31) = 1.25 |  |
| **Subjective Social Status** | 6.36± 1.77 | 5.96 ± 1.86 | 6.61 ± 1.74 | 5.83 ± 1.79 | F(3, 204.99) = 5.08^**^ | AS>MS^*^; IR<AA^*^; AA>MS^*^ |
| **Subjective Health** | 6.22 ± 2.29 | 4.35 ± 2.14 | 6.37 ± 2.54 | 4.63 ± 2.24 | F(3, 205.64) = 40.24^***^ | AS>IR^***^; AS>MS^***^; IR<AA^***^; AA>MS^***^ |
| **Perceived Stress** | 20.00 ± 6.97 | 26.30 ± 5.63 | 18.80 ± 7.35 | 25.40 ± 6.12 | F(3, 226.41) = 62.85^***^ | AS<IR^***^; AS<MS^***^; IR>AA^***^; AA<MS^***^ |
| **Study Satisfaction** | 7.80 ± 1.94 | 7.08 ± 2.25 | 8.19 ± 1.83 | 7.06 ± 2.15 | F(3, 202.9) = 9.17^***^ | AS>IR^**^; AS>MS^**^; IR<AA^***^; AA<MS^***^ |
| **Weekly Study Workload**, *hours* | 35.20 ± 17.20 | 37.90 ± 20.00 | 35.60 ± 18.40 | 40.60 ± 22.00 | F(3, 199.24) = 2.37 |  |
| **Study Engagement** | 3.06 ± 1.07 | 2.64 ± 1.06 | 3.30 ± 1.13 | 2.59 ± 1.07 | F(3, 204.89) = 13.19^***^ | AS>IR^***^; AS>MS^***^; IR<AA^***^; AA>MS^***^ |
| **Sleep Duration***, hours* | 7.38 ± 0.88 | 6.86 ± 1.08 | 7.15 ± 1.11 | 6.33 ± 1.17 | F(3, 196.2) = 31.49^***^ | AS>IR^***^; AS<MS^***^; IR>MS^**^; AA>MS^***^ |
| **Sleep Latency***, minutes* | 29.20 ± 27.70 | 47.00 ± 32.30 | 7.79 ± 4.44 | 41.40 ± 30.90 | F(3, 257.19) = 257.89^***^ | AS<IR^***^; AS>AA^***^; AS<MS^**^; IR>AA^***^; AA<MS^***^ |
| **PSQI Global Score** | 5.49 ± 2.15 | 9.43 ± 2.42 | 3.46 ± 1.81 | 9.34 ± 2.63 | F(3, 204.36) = 217.19^***^ | AS<IR^***^; AS>AA^***^; AS<MS^***^; IR>AA^***^; AA<MS^***^ |
| **PSQI Component Scores** |  |  |  |  |  |  |
| Subjective Sleep Quality | 1.15 ± 0.60 | 1.70 ± 0.60 | 0.83 ± 0.76 | 1.71 ± 0.64 | F(3, 201.76) = 58.85^***^ | AS<IR^***^; AS>AA^***^; AS<MS^***^; IR>AA^***^; AA<MS^***^ |
| Sleep Latency | 1.32 ± 0.90 | 2.08 ± 0.80 | 0.00 ± 0.00 | 1.80 ± 0.88 | – |  |
| Sleep Duration | 0.17 ± 0.40 | 0.55 ± 0.64 | 0.40 ± 0.56 | 0.98 ± 0.90 | F(3, 188.86) = 41.42^***^ | AS<IR^***^; AS<AA^**^; AS<MS^***^; IR<MS^***^; AA<MS^***^ |
| Habitual Sleep Efficiency | 0.42 ± 0.67 | 0.98 ± 1.02 | 0.09 ± 0.32 | 0.99 ± 0.95 | F(3, 218.43) = 58.22^***^ | AS<IR^***^; AS>AA^***^; AS<MS^***^; IR>AA^***^; AA<MS^***^ |
| Sleep Disturbances | 0.97 ± 0.17 | 2.00 ± 0.00 | 0.59 ± 0.56 | 0.99 ± 0.10 | – |  |
| Use of Sleep Medications | 0.00 ± 0.00 | 0.14 ± 0.42 | 0.00 ± 0.00 | 0.90 ± 0.77 | – |  |
| Daytime Functionality | 1.45 ± 0.70 | 1.98 ± 0.62 | 1.55 ± 0.81 | 1.98 ± 0.74 | F(3, 204.49) = 38.41^***^ | AS<IR^***^; AS<MS^***^; IR>AA^***^; AA<MS^***^ |

*Notes.* ^*^*p* < .05, ^**^*p* < .01, ^***^*p* < .001. Values are expressed as mean ± standard deviation. PSQI components range from 0 to 3, with higher values indicating more maladaptive sleep quality outcomes. “ – “ = For some variables, ANOVA results could not be computed due to zero or near-zero variance in one or more groups, which violates the assumptions required for variance-based tests.
